# Effect of DNA methylation on the osteogenic differentiation of mesenchymal stem cells: concise review

**DOI:** 10.3389/fgene.2024.1429844

**Published:** 2024-07-02

**Authors:** Zhihao Lai, Qing Shu, Yue Song, Ao Tang, Jun Tian

**Affiliations:** ^1^ Department of Rehabilitation Medicine, Zhongnan Hospital of Wuhan University, Wuhan, China; ^2^ College of Sports Medicine, Wuhan Sports University, Wuhan, China

**Keywords:** mesenchymal stem cells (MSCs), DNA methylation, DNA methyltransferases (DNMTs), Ten-eleven translocation family proteins (TETs), osteogenic differentiation, signaling pathway

## Abstract

Mesenchymal stem cells (MSCs) have promising potential for bone tissue engineering in bone healing and regeneration. They are regarded as such due to their capacity for self-renewal, multiple differentiation, and their ability to modulate the immune response. However, changes in the molecular pathways and transcription factors of MSCs in osteogenesis can lead to bone defects and metabolic bone diseases. DNA methylation is an epigenetic process that plays an important role in the osteogenic differentiation of MSCs by regulating gene expression. An increasing number of studies have demonstrated the significance of DNA methyltransferases (DNMTs), Ten-eleven translocation family proteins (TETs), and MSCs signaling pathways about osteogenic differentiation in MSCs. This review focuses on the progress of research in these areas.

## 1 Introduction

Friedenstein et al. first discovered mesenchymal stem cells (MSCs) in bone marrow (Friedenstein et al., 1970). MSCs originate from embryonic stem cells and can differentiate into various cell types, including osteoblasts, chondrocytes, adipocytes, and other cell lineages (da Silva Meirelles et al., 2006; Rastegar et al., 2010). It can be extracted from adult tissues, including bone marrow, umbilical cord blood, adipose tissue, and tooth tissue (Hu et al., 2018). In an aging society, severe bone defects caused by trauma, infection, tumors, and skeletal developmental abnormalities are increasingly challenging treatments in orthopedics and trauma surgery. The development of bone healing and regeneration strategies is of great significance for improving patient function and quality of life (Cancedda et al., 2007). MSCs are considered promising seed cells for bone tissue engineering in bone healing and regeneration due to their self-renewal, multiple differentiation, and immunomodulatory properties (Seong et al., 2010). In normal bones, the differentiation of MSCs into osteoblasts is regulated by multiple signaling pathways, such as bone morphogenetic proteins (BMPs), Notch, Hedgehog, neuroepidermal growth factor like protein 1 (NELL-1), wnt signaling, growth factors, and MAPK signaling pathways (Montecino et al., 2020; Ahmadi et al., 2022; Thomas and Jaganathan, 2022). The transcription factors Runx2 and Osterix (Osx) are the main regulatory factors of osteogenesis, inducing osteogenic lineage commitment and differentiation of MSCs through interactions with various signaling pathways (Yang et al., 2004). There is evidence to suggest that DNA methylation plays an important role in regulating the molecular pathways involved in the osteogenesis of MSCs (Levi and Longaker, 2011).

DNA methylation is a part of epigenetics, controlled by enzymes from the DNA methyltransferases (DNMTs) family. It modifies DNA without changing its sequence, which affects gene expression (Moore et al., 2013). DNA methylation plays an important role in genomic imprinting, tissue-specific gene expression, and X chromosome inactivation (Walewska et al., 2023). It also regulates stem cell maintenance and differentiation by activating or inhibiting certain genes (Kouzarides, 2007). Research has shown that DNA methylation status is associated with the osteogenic ability of MSCs. DNA demethylation has been shown to play a beneficial role in the osteogenesis of MSCs (Yan et al., 2014). During the osteogenic differentiation process of MSCs, the expression of osteogenic specific transcription factor genes, RUNX2 and OSX, increases, accompanied by a decrease in their DNA methylation (Farshdousti Hagh et al., 2012; Xu et al., 2017). However, there are also opposing reports indicating that DNA demethylation impairs the osteogenic potential of MSCs (Xin et al., 2020). The role of DNA methylation in regulating the osteogenic differentiation of MSCs is not fully understood. Further research on the mechanism of DNA methylation regulating the osteogenic differentiation of MSCs can help us gain a more comprehensive understanding of the basic biological processes involved in stem cell differentiation and cell fate determination. This can allow us to fully utilize the characteristics of MSCs as seed cells to treat related diseases. This article reviews the progress made in understanding the mechanism of DNA methylation on the osteogenic differentiation of MSCs from the viewpoint of enzymes mediating DNA methylation and their impact on downstream signaling pathways related to osteogenic differentiation.

## 2 Overview of DNA methylation

Epigenetic phenomena play a crucial role in cellular regulation and are significant factors in comprehending complex human diseases. Among the extensively studied epigenetic mechanisms, DNA methylation holds a prominent position (Kriukienė et al., 2024). DNA methylation, in the field of epigenetics, usually refers to 5’methylcytosine (5mC) where the fifth carbon atom of cytosine is added with a methyl group to form 5-methylcytosine (Patil et al., 2014). Most 5-methylcytosine is located directly in front of the 5′guanine residue, known as CpG methylation, in most mammalian cells. Non-CpG methylation can occur on CHG (H = A, C, or T) and CHH sites (Sarkies, 2022). CpG islands (CGIs) are high CpG density regions spread throughout the mammalian genome, with a length of 300-3000 bp, primarily located in the promoter and first exon/5′- untranslated region of the gene. Approximately 70% of human genes have promoter regions that overlap with these regions, making DNA methylation capable of affecting mRNA levels without changing the gene sequence (Luczak and Jagodziński, 2006; Saxonov et al., 2006). DNA methylation is closely related to gene transcriptional inhibition. It is generally believed that DNA methylation achieves negative regulation of gene expression through two pathways: direct inhibition of gene transcription by interfering with transcription factors binding to gene promoters (or other cis-regulatory sequences) and indirect inhibition of gene transcription by recruiting transcription inhibitors through 5mC binding proteins (Greenberg and Bourc’his, 2019). However, DNA methylation often occurs within the gene body, between the transcription start and end sites. In contrast to the effect of promoter methylation, genomic methylation is usually positively correlated with gene expression (Neri et al., 2017). The primary purpose of DNA methylation is genome defense, which maintains the silence of transposable elements to prevent transposition from affecting the stability of the host genome. As species evolve, DNA methylation gradually participates in other biological processes, making its functions increasingly complex (Jones, 2012).

**FIGURE 1:**
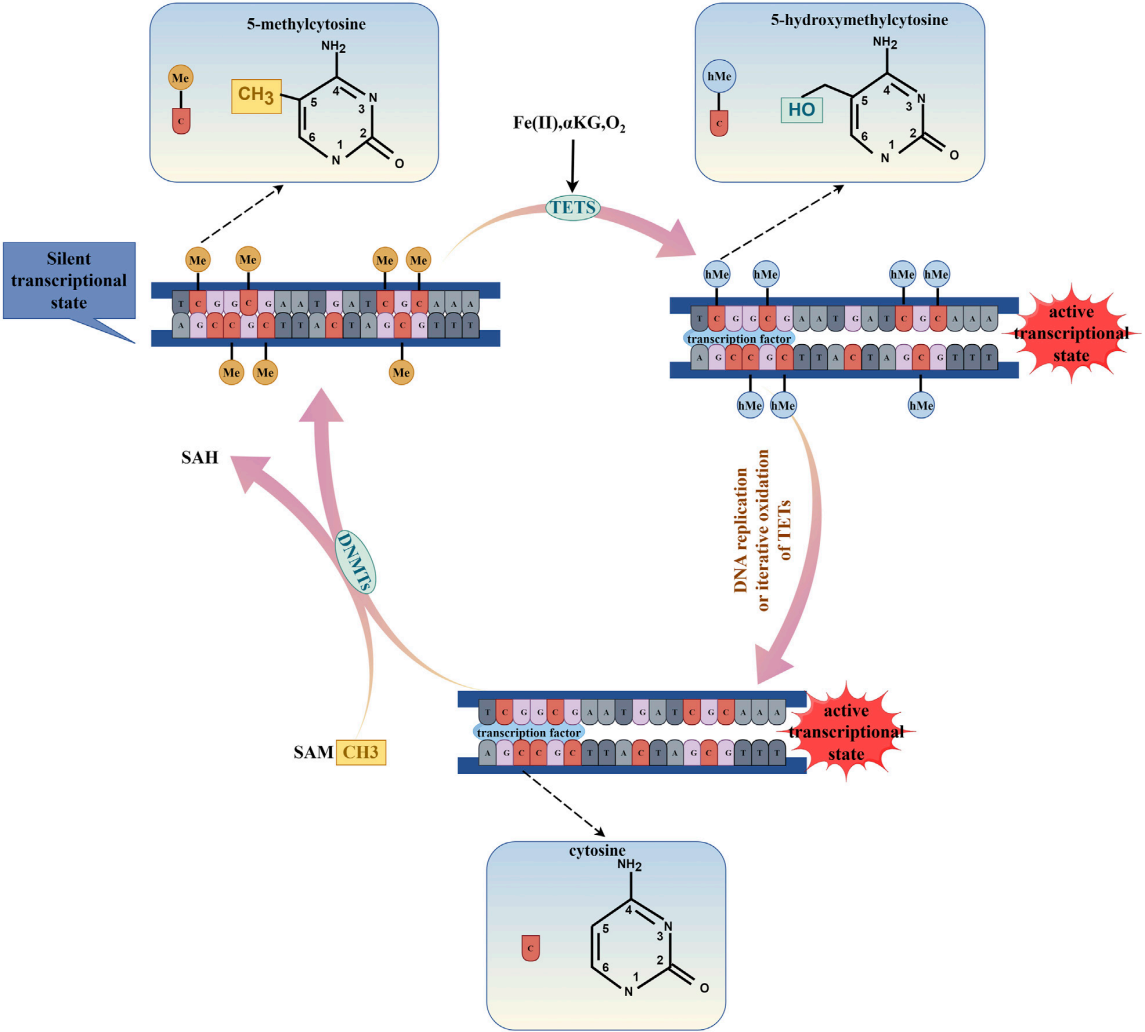
The process of DNA methylation and DNA demethylation. (By Figdraw.). DNA methylation is enzymatically catalyzed by DNMTs, which utilize S-adenosylmethionine (SAM) as a methyl donor to transfer a methyl group to the fifth carbon atom (C5) of the cytosine ring, thereby leading to the transcriptional repression of genes. Conversely, DNA demethylation is facilitated by the TETs enzymes through a process of iterative oxidation, which requires the presence of Fe(II), α-KG, and O_2_. Additionally, DNA demethylation can occur during DNA replication. This demethylation process results in the transcriptional activation of genes.

DNA methylation is catalyzed by DNMTs, using S-adenosylmethionine as a methyl donor to transfer the methyl group to the fifth carbon atom (C5) of cytosine (Figure 1). According to the different modes of action of methyltransferases, DNMTs can be mainly divided into two categories: one is initiating DNA methyltransferases (*de novo* DNMTs), and the other is maintenance DNA methyltransferases (maintenance DNMTs) (Goll and Bestor, 2005). DNMT1 is responsible for maintaining DNA methylation. It binds to partially methylated CpG sites and spreads the methylation pattern to newly synthesized DNA. DNMT1’s activity is higher at partially methylated sites compared to unmethylated sites, indicating its role in maintenance methylation (Pradhan et al., 1999). DNMT1 is expressed in cells that are actively dividing and is associated with DNA replication. When the DNMT1 gene is knocked out in mouse embryonic stem cells, the genome-wide DNA methylation decreases significantly (Leonhardt et al., 1992; Li et al., 1992; Lei et al., 1996). DNMT3 family members, DNMT3A, DNMT3B, and DNMT3L are responsible for *de novo* methylation, which establishes new methylation patterns on unmethylated DNA (Okano et al., 1998). DNMT3A and DNMT3B are expressed during embryonic development, consistent with the timing of *de novo* methylation (Okano et al., 1998). In mouse embryonic stem cells and embryos, DNMT3A and DNMT3B double knockout prevents *de novo* methylation without affecting maintenance methylation (Okano et al., 1999). DNMT3L lacks catalytic activity but binds to DNMT3A and DNMT3B, enhancing their catalytic activity (Veland et al., 2019). DNMTs are essential for maintaining genomic integrity and silencing specific sequences, including imprinted genes, inactive X-chromosome genes, and transposons. Loss of DNMTs function may lead to chromosomal instability and tumor progression (Robertson, 2005).

DNA demethylation is a process that is mediated by proteins belonging to the Ten Eleven Translation Family (TETs), which include TET1, TET2, and TET3 in mammals. The process involves oxidative modification of 5-methylcytosine (5mC) to produce 5-hydroxymethylcytosine (5hmC), 5-formylcytosine (5fC), and 5-carboxylcytosine (5caC) that ultimately lead to DNA demethylation and promote gene transcription (Ooi and Bestor, 2008; Wang L. et al., 2022). TETs are crucial for stem cell differentiation, and the loss of TET1/2/3 can impair the differentiation of mouse embryonic stem cells (Dawlaty et al., 2014). Active demethylation intermediates can act as heritable epigenetic markers. 5hmC is involved in gene regulation and is predominantly present in actively transcribed genes. It binds to specific regulatory proteins capable of transmitting regulatory functions (Bachman et al., 2014). 5fC and 5caC interact with many proteins, including TDG, p53, DNA repair factors, chromatin remodeling factors, and forkhead box TFs. The presence of 5fC and 5caC in template DNA strands can temporarily suspend and decrease the elongation of RNA polymerase II (pol II) *in vivo* and *in vitro*. Controlled deposition of 5fC and 5caC can fine-tune the elongation of specific genes and indirectly affect gene expression (Rasmussen and Helin, 2016).

## 3 The role of DNMTs in osteogenic differentiation of MSCs

There is increasing evidence that DNA methylation, facilitated by various DNMTs, plays a crucial role in bone biology (Husain and Jeffries, 2017). Studies have shown that abnormal expression of DNMTs can influence the expression of genes and pathways that are related to osteoblasts and osteoclasts, thereby affecting bone remodeling (Liu et al., 2016). The process of MSCs differentiation requires the gradual loss of stem cell features and the acquisition of specific cell line or tissue-specific cellular identity, which is achieved through regulatory mechanisms such as epigenetic modification. The methylation of osteogenic-related gene promoters or enhancers regulates the osteogenic process of MSCs, while the DNMTs family mediates DNA methylation (Weissbein et al., 2017; Wang X. et al., 2023). In fact, OCT4 enhances the transcription of DNMTs genes, leading to an increase in p21 promoter methylation, promoting the self-renewal ability of human hair follicular mesenchymal stem cells (hHFMSCs), and reversing aging and osteogenic differentiation ability (Lu et al., 2019). Moreover, the expression of DNMT3A and DNMT3B increased in three-dimensional spherical osteogenic differentiation cultures, and the significant downregulation of DNMT3B in two-dimensional monolayer osteogenic differentiation culture affected the osteogenic potential of Dental pulp stem cells (DPSCs). However, the expression of DNMT1 remained unchanged in both two-dimensional monolayer and three-dimensional spherical osteogenic differentiation cultures (Raik et al., 2022). By gaining a deeper understanding of DNMTs that regulate gene expression, we can better comprehend the structure and osteogenic differentiation functional behavior of MSCs, and improve treatment methods based on MSCs.

### 3.1 The role of maintenance methyltransferase (DNMT1) in osteogenic differentiation of MSCs

DNMT1 is a vital component in both developmental and adult somatic cells, which ensures cell proliferation and survival (Jurkowska et al., 2011). It plays a crucial role in maintaining CpG methylation, regulating chromatin modification and remodeling in stem cell renewal and differentiation (Robert et al., 2003; Tsumura et al., 2006). Additionally, the downregulation of DNMT1 can lead to passive DNA demethylation, which can enhance the spontaneous differentiation of MSCs (Zhang et al., 2016). Many studies have reported the role of DNMT1 in regulating the directional osteogenic lineage differentiation of MSCs (Table 1).

**TABLE 1:** The role of DNMT1 in osteogenic differentiation of MSCs.

Cell type	Gene loci regulated by DNMT1	Brief outcomes	The effect of DNMT1 on osteogenic differentiation potential	References
mouse BMSCs	OPG	LncRNA SNHG1 interacts with PTBP1→DNMT1↑→OPG↓→osteogenic differentiation↓	↓	Kang et al. (2015)
human DFSCs	HOXA2	LncRNA HOAIRM1→DNMT1↓→ HOXA2↑→ osteogenic differentiation↑	↓	Karasik et al. (2002)
mouse BMSCs	Notch1	miR-29b→DNMT1↓→Notch1↑→osteogenic differentiation↓	↑	Kato et al. (2009)
human BMSCs	LncRNA MEG3	DNMT1→ LncRNA-MEG3↓→BMP4↓→osteogenic differentiation↓	↓	Kim et al. (2013b)
pig BMSCs, human BMSCs	OCT4, NANOG	DNMT1↓→OCT4, NANOG↑→osteogenic differentiation↑	↓	Kriukienė et al. (2024)
human BMSCs	P16, p21, developmental and lineage differentiation genes	OCT4, NANOG→ DNMT1↑→P16, p21, developmental and lineage differentiation genes↓→osteogenic differentiation↓	↓	Lee (2012)
human ADSCs	PREF1, p21, p27	DNMT1↓→ p53, PREF1↓→p21, p27↑→ osteogenic differentiation↓	↑	Lei et al. (1996)
human PDLSCs	CALCA	AGEs→ DNMT1↑→CALCA↓→osteogenic differentiation↓	↓	Levi and Longaker (2011)
rat ADSCs, mouse ADSCs	undefined	AGEs→ DNMT1, DNMT3A, DNMT3B↑→5-mC↑→Wnt singnaling pathway↓→osteogenic differentiation↓	↓	(Li et al., 1992; Li et al., 2019a)
human ADSCs	Notch1, Notch2	SIRT6→ DNMT1↓→Notch1, Notch2↑→osteogenic differentiation↑	↓	Li et al. (2021a)
human BMSCs	p16/p21	RB protein binds to c-JUN → DNMT1↑→p16/p21↓→osteogenic differentiation↑	↑	Li et al. (2013)
human HFMSCs	p21	OCT4→ DNMT1, DNMT3A, DNMT3B↑→p21↓→osteogenic differentiation↑	↑	Husain and Jeffries (2017)
C3H10T1/2 cells	Rora, Fgfr2, Ihh	DNMT1↓→Rora, Fgfr2, Ihh↑→osteogenic differentiation↑	↓	Jiang et al. (2019)

Note: ↑: Promoting; ↓: Inhibiting; →: Regulating.

Abbreviation: DNMTs, DNA, methyltransferases; DNMT1, DNA, methyltransferase 1; MSCs, mesenchymal stem cells; BMSCs, bone marrow mesenchymal stem cells; OPG, osteoprotegerin; PTBP1, polypyrimidine tract-binding protein 1; HOXA2, Homeobox A2; DFSCs, dental follicle stem cells; BMP4, Bone Morphogenetic Protein 4; ADSCs, Adipose Derived Stem Cells; NANOG, nanog homeobox; PREF1, Delta Like Non-Canonical Notch Ligand 1; PDLSCs, periodontal ligament stem cells; CALCA, calcitonin related polypeptide alpha; AGEs, advanced glycation end products; 5-mC, 5-methylcytosine; SIRT6, Sirtuin 6; RB, retinoblastoma protein; HFMSCs, hair follicle-derived mesenchymal stem cells; Rora, RAR, Related Orphan Receptor A; Fgfr2, Fibroblast Growth Factor Receptor 2; Ihh, Indian Hedgehog.

**FIGURE 2:**
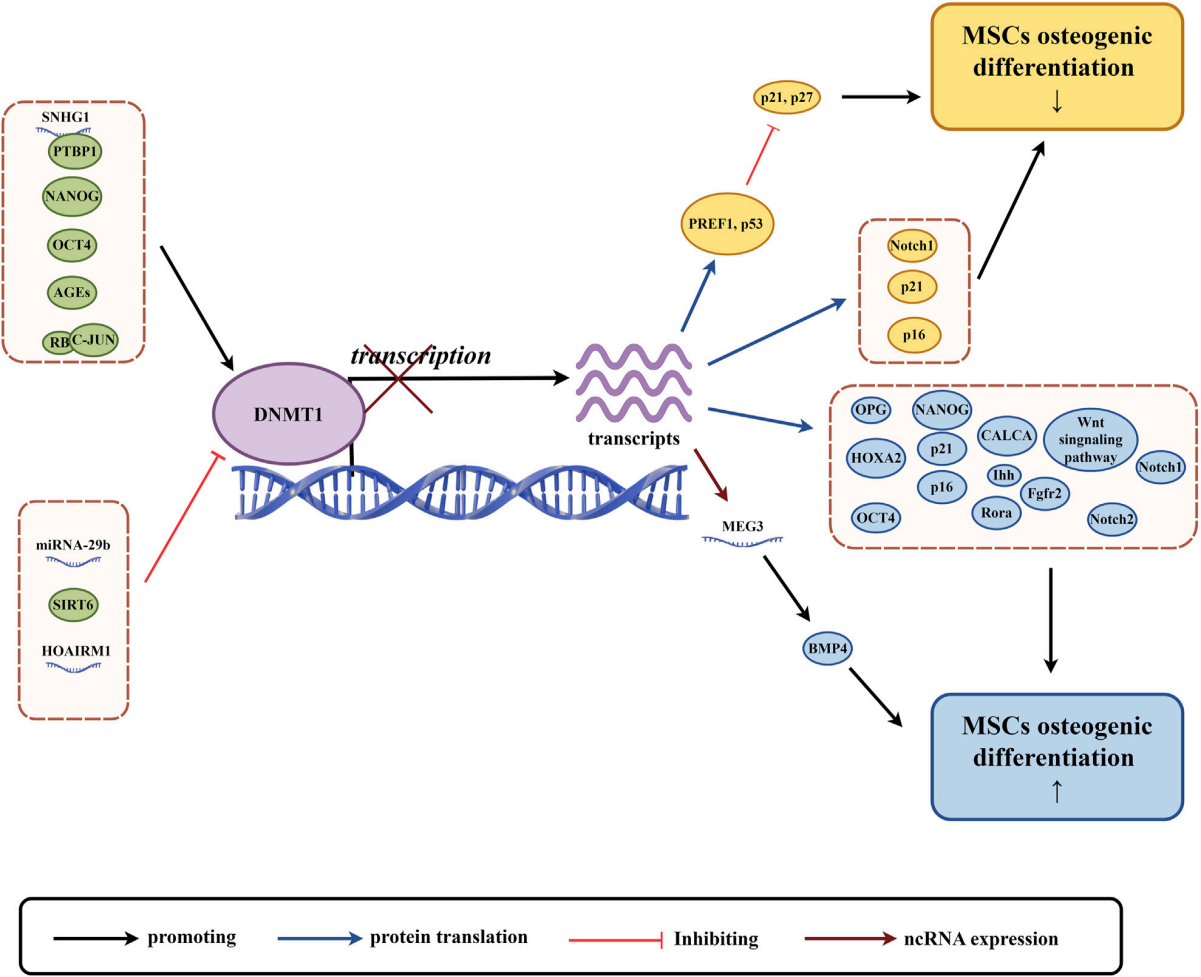
The role of DNMT1 in osteogenic differentiation of MSCs. (By Figdraw.). The expression and activity of DNMT1 are regulated by various factors, including non-coding RNAs and proteins (highlighted in green circles). These factors influence DNA methylation and subsequent gene transcription and translation. This regulation affects the expression of proteins related to osteogenic differentiation of MSCs, the Wnt signaling pathway, and non-coding RNAs, with yellow circles indicating factors associated with the downregulation of MSCs osteogenic differentiation, and blue circles indicating factors associated with the upregulation of MSCs osteogenic differentiation.

DNMT1 plays a crucial role in the process of osteogenic differentiation of MSCs (Figure 2). Studies have shown that the expression of DNMT1 is reduced during the differentiation of bone marrow mesenchymal stem cells (BMSCs) or osteoprogenitor cells into osteoblasts. However, in humans and mice with age-related bone loss, the expression of DNMT1 increases in osteoprogenitor cells (Tao et al., 2022). Similarly, another study found that the expression level of DNMT1 decreased after osteogenic differentiation of human amniotic fluid-derived mesenchymal stem cells (AF-MSCs) (Glemžaitė and Navakauskienė, 2016). During the process of osteogenic differentiation of MSCs, DNMT1 is downregulated, which may be related to the upregulation of osteogenic lineage differentiation genes. On the other hand, DNMT1 methylates osteogenic differentiation-related genes and inhibits their expression (Cakouros and Gronthos, 2020).

There is a relationship between DNMT1 (a protein that helps modify DNA) and non-coding RNA (RNA that does not produce proteins), which influences the way in which genomic methylation patterns are established and abnormal DNA methylation is regulated (Di Ruscio et al., 2013). This relationship can affect the process of creating bone cells from adult stem cells in various ways. For example, a type of non-coding RNA called LncRNA SNHG1 can activate the expression of DNMT1, which in turn can lead to the hypermethylation of a gene called osteoprotegerin (OPG) and the inhibition of OPG expression. This can damage the process of creating bone cells from mouse BMSCs (Yu X. et al., 2022). Conversely, another type of non-coding RNA known as LncRNA HOTAIRM1 can inhibit DNMT1 expression, which leads to low methylation of a gene called HOXA2 and an increase in HOXA2 expression. This promotes the process of creating bone cells from human dental follicle stem cells (hDFSCs) (Chen et al., 2020). In mice with lupus disease, a deficiency in a protein called Fas can lead to higher levels of miR-29b, a type of RNA. This can decrease the expression of DNMT1 in MRL/lpr BMSCs and contribute to low methylation of the Notch1 promoter, which can impair the process of creating bone cells (Liu et al., 2015). LncRNA, like any other protein coding gene, is regulated by the same epigenetic mechanism, and DNMT1 can regulate its expression (Lee, 2012).Finally, the expression of a non-coding RNA called LncRNA MEG3 is inhibited in children with aplastic anemia (AA) due to high levels of DNMT1 expression, which can lead to lower levels of bone morphogenetic protein 4 (BMP4) transcriptional activity and impair the process of creating bone cells from BMSCs in AA patients (Li H. et al., 2021). Overall, the relationship between DNMT1 and non-coding RNA may play an important role in regulating the process of creating bone cells from MSCs, and further research is necessary to understand this relationship more completely.

The OCT4, SOX2, and NANOG genes are responsible for regulating the self-renewal and pluripotency of stem cells. They form a transcriptional network that is essential for the differentiation potential of MSCs (Hyslop et al., 2005; Liu et al., 2009). DNMT1 is a protein that can affect the methylation of OCT4 and NANOG promoters and regulate their expression. This regulation can alter the tripartite differentiation of pig BMSCs and human BMSCs, making DNMT1 an early indicator and regulator of stem cell potential (Li K. et al., 2020). NANOG and OCT4, in turn, can bind to the promoter of DNMT1 and enhance its expression, indirectly regulating gene expression (Tsai et al., 2012). Recent research suggests that upregulation of DNMT1 can maintain DNA methylation of p16 and p21, as well as genes related to development and lineage differentiation. This maintenance inhibits gene expression, maintains cell proliferation, and undifferentiated status, and decreases osteogenic differentiation ability of human BMSCs (hBMSCs) (Tsai et al., 2012). On the other hand, downregulation of NANOG in human adipose-derived stem cells (hADSCs) can cause a decrease in DNMT1 protein expression, leading to decreased methylation of PREF1, p21, and p27. This decrease results in an upregulation of p21 and p27, causing hADSCs to stagnate in the G0/G1 phase. This stagnation ultimately results in decreased differentiation ability and impaired osteogenic differentiation function (Pitrone et al., 2019).

Advanced glycation end products (AGEs) are harmful pathogenic factors that are closely related to complications of diabetes. AGEs cause oxidative stress by cross-linking extracellular matrix, which damages the proliferation and osteogenic differentiation of MSCs and increases osteoclast production (Asadipooya and Uy, 2019). During the process of osteogenic induction of human periodontal ligament mesenchymal stem cells (hPDLSCs), AGEs upregulate the expression of advanced glycosylation end product-specific receptor (RAGE) and DNMT1, and increase the expression of calcitonin-related peptides α (CALCA). The methylation level of the CALCA promoter is increased, and the downregulation of CALCA expression impairs the osteogenic differentiation ability of hDPLSCs (Wang Q. N. et al., 2022). When rat ADSCs and mouse ADSCs were cultivated in media containing AGEs, high levels of DNMTs (DNMT1, DNMT3A, and DNMT3B) in cells led to an increase in global 5-mC. The classical Wnt signaling pathway and osteogenic differentiation were inhibited as a result (Zhang et al., 2018; Li Y. et al., 2020).

In addition to the research reports mentioned above, DNMT1 can also be influenced by other factors that affect the osteogenic differentiation of MSCs. The nicotinamide adenine dinucleotide (NAD) dependent deacetylase SIRT6 deacetylates DNMT1, causing protein instability and preventing abnormal DNA methylation of Notch1 and Notch2, resulting in their transcriptional upregulation and promoting osteogenic differentiation of hADSCs (Jia et al., 2021). Retinoblastoma (RB) protein binds to c-JUN to increase the expression of DNMT1, methylate the promoter of p16/p21 cyclin dependent kinase inhibitor gene, reduce its expression, inhibit aging of hBMSCs, increase differentiation potential and bone repair ability (Lin et al., 2014).

### 3.2 The role of *De Novo* DNA methyltransferases (DNMT3A, DNMT3B, DNMT3L) in osteogenic Differentiation of MSCs

**FIGURE 3:**
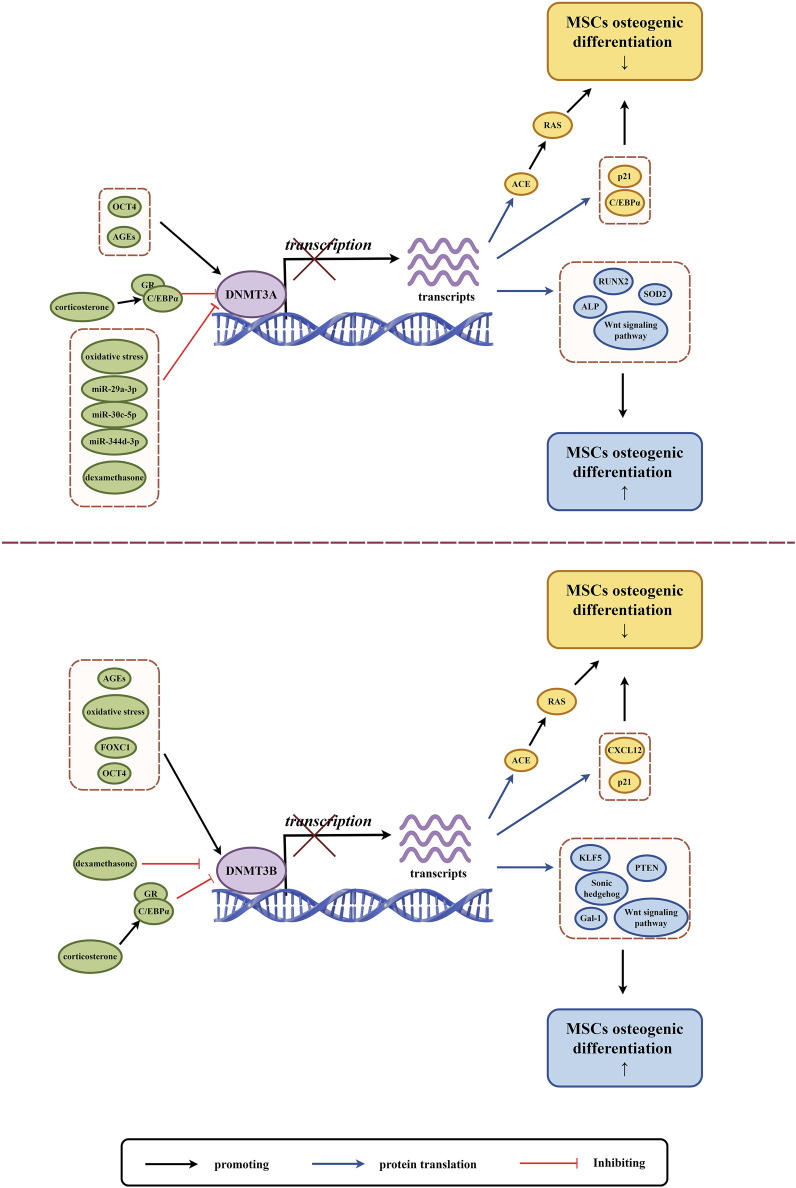
The role of DNMT3A and DNMT3B in osteogenic differentiation of MSCs. (By Figdraw.). The expression and activity of DNMT3A and DNMT3B are regulated by various factors (highlighted in green circles), which influence DNA methylation and subsequent gene transcription and translation. This regulation impacts the expression of proteins and the Wnt signaling pathway related to MSCs osteogenic differentiation, where yellow circles indicate factors associated with the downregulation of MSCs osteogenic differentiation, and blue circles indicate factors associated with the upregulation of MSCs osteogenic differentiation.

DNMT3A and DNMT3B are genes that are highly active in the early stages of mammalian embryos. However, their activity decreases as cells differentiate (Zhang and Xu, 2017). These genes are responsible for adding methyl groups to DNA, a process known as DNA methylation. This process plays a significant role in the differentiation of MSCs (Table 2) (Figure 3) (Busque et al., 2018).

**TABLE 2:** The role of DNMT3 family members in osteogenic differentiation of MSCs.

DNMT3 type	Cell type	Gene loci regulated by DNMT3	Brief outcomes	The effect of DNMT3 on osteogenic differentiation potential	References
DNMT3A	human BMSCs	ALP, RUNX2	oxidative stress→DNMT3A↓→ALP, RUNX2↑→osteogenic differentiation↑	↓	Liu et al. (2009)
	human ADSCs	SOD2	miR-29a-3p, miR-30c-5p→DNMT3A↓→SOD2↑→osteogenic differentiation↑	↓	Lyu et al. (2019)
	mouse MBMSCs	undefined	miR-344d-3p↓→DNMT3A↑→osteogenic differentiation↓	↓	Marofi et al. (2020)
	rat BMSCs	C/EBPα	dexamethasone→DNMT3A, DNMT3B→C/EBPα↑→osteogenic differentiation↓	↓	Marofi et al. (2019b)
	rat BMSCs	ACE	corticosterone→GR-C/EBPα-DNMT3A/DNMT3B→ACE↑→RAS↑→osteogenic differentiation↓	↑	Maroni et al. (2012)
	rat ADSCs, mouse ADSCs	undefined	AGEs→ DNMT1, DNMT3A, DNMT3B↑→5-mC↑→Wnt singnaling pathway↓→osteogenic differentiation↓	↓	(Li et al., 1992; Li et al., 2019a)
	human HFMSCs	p21	OCT4→ DNMT1, DNMT3A, DNMT3B↑→p21↓→osteogenic differentiation↑	↑	Husain and Jeffries (2017)
DNMT3B	mouse BMSCs	KLF5	oxidative stress→DNMT3B↑→ KLF5↓→osteogenic differentiation↓	↓	Liu et al. (2015)
	rat BMSCs	C/EBPα	dexamethasone→DNMT3A, DNMT3B→C/EBPα↑→osteogenic differentiation↓	↓	Marofi et al. (2019b)
	rat BMSCs	ACE	corticosterone→GR-C/EBPα-DNMT3A/DNMT3B→ACE↑→RAS↑→osteogenic differentiation↓	↑	Maroni et al. (2012)
	human UMSCs	undefined	DNMT3B↓→osteogenic differentiation↓	↑	McBeath et al. (2004)
	mouse BMSCs	Gal-1	DNMT3B→ Gal-1↓→osteogenic differentiation↓	↓	Monroe et al. (2012)
	mouse BMSCs	Sonic hedgehog	DNMT3B↑→Sonic hedgehog↓→osteogenic differentiation↓	↓	Montecino et al. (2020)
	human BMSCs	CXCL12	FOXC1→DNMT3B↑→CXCL12↓→osteogenic differentiation↑	↑	Moore et al. (2013)
	human BMSCs, human DPMSCs	PTEN	DNMT3B→PTEN↓→osteogenic differentiation↓	↓	Moosa and Wollnik (2016)
	rat ADSCs, mouse ADSCs	undefined	AGEs→ DNMT1, DNMT3A, DNMT3B↑→5-mC↑→Wnt singnaling pathway↓→osteogenic differentiation↓	↓	(Li et al., 1992; Li et al., 2019a)
	human HFMSCs	p21	OCT4→ DNMT1, DNMT3A, DNMT3B↑→p21↓→osteogenic differentiation↑	↑	Husain and Jeffries (2017)
DNMT3L	mouse BMSCs	undefined	DNMT3L↓→osteogenic differentiation↓	↑	Mori et al. (2014)

Note: ↑: Promoting; ↓: Inhibiting; →: Regulating.

Abbreviation: MSCs, mesenchymal stem cells; DNMTs, DNA, methyltransferases; DNMT3A, DNA, methyltransferase 3A; DNMT3B, DNA, methyltransferase 3B; KLF5, zinc finger transcription factor krüppel-like factor 5; BMSCs, bone marrow mesenchymal stem cells; ALP, alkaline phosphatase; RUNX2, RUNX, Family Transcription Factor 2; ADSCs, Adipose Derived Stem Cells; SOD2, Superoxide Dismutase 2; MBMSCs, mandibular bone marrow mesenchymal stem cells; C/EBPα, CCAAT, enhancer binding protein alpha; ACE, Angiotensin I Converting Enzyme; GR, glucocorticoid receptor; UMSCs, Umbilical Cord Mesenchymal Stem Cells; Gal-1, Galectin-1; CXCL12, C-X-C Motif Chemokine Ligand 12; FOXC1, Forkhead Box C1; DPMSCs, Dental pulp mesenchymal stem cells; PTEN, phosphatase and tensin homolog; AGEs, advanced glycation end products; 5-mC, 5-methylcytosine; HFMSCs, hair follicle-derived mesenchymal stem cells; DNMT3L, DNA, methyltransferase 3L.

MSCs with varying osteogenic differentiation potentials exhibit different levels of *de novo* DNA methyltransferases. MSCs with low osteogenic potential in the PDLCs express higher levels of DNMT3A and lower levels of RUNX2 compared to high osteogenic potential PDLCs (Ferreira et al., 2022). As a stem gene of human induced pluripotent stem cell-derived mesenchymal stem cells (iPSC-MSCs), DNMT3B expression level is lower than that of BMSCs, and it also shows insufficient osteogenic differentiation ability (Kang et al., 2015). MSCs derived from the subchondral bone of the rat condyle (SMSCs) have higher expression of DNMT3B and significantly higher mineralization and osteogenic differentiation potential compared to MSCs derived from the tibia (TMSCs) and mandibular ramus bone marrow (MMSCs) (Wang Y. et al., 2023). Osteogenesis imperfecta (OI) mice with ADSCs express high levels of DNMT3A compared to wild-type mice with ADSCs, and their osteogenic differentiation ability and collagen secretion are significantly impaired (Shao et al., 2023). Differentially expressed DNMTs may reflect the differences in epigenetic regulation between different subgroups of MSCs, and this regulation may be one of the key factors in maintaining the characteristics of different MSCs subgroups.

Oxidative stress (OS) refers to an imbalance between the creation and removal of reactive oxygen species (ROS), which leads to a build-up of ROS in the body. This condition is caused by various factors such as ischemia, hypoxia, and inflammation (Muinos-López et al., 2016). Excessive ROS can harm nucleic acids, proteins, and lipids. Severe oxidative stress can inhibit cell survival, proliferation, and differentiation, and it can also inhibit osteogenic differentiation (Feng et al., 2013). Studies have shown that OS can induce changes in DNMTs and regulate genomic DNA methylation (O'Hagan et al., 2011; Li et al., 2014). OS mediates high methylation of zinc finger transcription factor krüppel-like factor 5 (KLF5) through DNMT3B, leading to a decrease in KLF5 expression and a decrease in association with β-catenin. This impairs the osteogenic differentiation of mouse BMSCs (Li L. et al., 2021). Liangping Li et al. established a new 3D cell culture model in which OS mediated low methylation of (Alkaline Phosphatase) ALP and RUNX2 promoters through DNMT3A downregulation, and upregulation of ALP and RUNX2 expression improved osteogenic differentiation of hBMSCs (Li L. et al., 2019).

As discussed previously, microRNAs (miRNAs) can affect DNA methylation and interfere with epigenetic mechanisms by altering the expression of DNA methylase or its co-proteins, which are intricately linked to osteoblast differentiation (Kim E. J. et al., 2013; Saviana et al., 2023). MiR-29a-3p and miR-30c-5p have an inhibitory function by binding directly to the DNMT3A 3′UTR, which reduces the methylation of CpG islands in the upstream regulatory region of the antioxidant gene superoxide dismutase 2 (SOD2). This leads to the restoration of SOD2 expression and alleviation of oxidative stress in hADSCs, improving mitochondrial function, cellular aging status, multi-directional differentiation potential of hADSCs and enhancing osteogenic differentiation ability (Jung et al., 2020). In the bone microenvironment of postmenopausal osteoporosis, the expression of miR-344d-3p in mouse mandibular BMSCs decreases. Due to the presence of two binding sites of miR-344d-3p in the 3′-UTR region of DNMT3A, an increase in DNMT3A leads to downregulation of osteogenic differentiation and upregulation of adipogenic differentiation in mouse mandibular BMSCs (Cao et al., 2023).

Glucocorticoids in normal concentrations promote osteoblast growth and stimulate the differentiation of MSCs into bone cells. However, at drug concentrations, glucocorticoids can cause apoptosis (cell death) of osteoblasts and bone cells, as well as hinder the differentiation and growth of bone progenitor cells (Han et al., 2019). Dexamethasone, for instance, blocks the binding of DNMT3A and DNMT3B to C/EBP alpha promoters, which can lead to an increase in CCAAT/enhancer α (C/EBP alpha) mRNA and protein levels *in vivo* and *in vitro*. High levels of C/EBP alpha can shift the fate of rat BMSCs towards adipocytes instead of osteoblasts (Li et al., 2013). In a study on prenatal exposure to caffeine (PCE), corticosterone was found to act through GR-C/EBP alpha, which in turn led to low methylation of angiotensin-converting enzyme (ACE). As a result, ACE expression increased and led to sustained activation of the RAS pathway, which inhibited osteogenic differentiation of rat BMSCs (Wen et al., 2019).

Wharton’s jelly derived MSCs (WJ-MSCs) obtained from human umbilical cord were treated with graphene oxide (GO) and were found to exhibit a decrease in DNMT3B expression after undergoing osteogenic induction (Hiew and Teoh, 2024). Interestingly, RNA interference was used to inhibit endogenous DNMT3B expression in human umbilical cord mesenchymal stem cells (hUMSCs), which resulted in impaired osteogenic differentiation of hUMSCs (Zhu et al., 2014). Depending on the intracellular and extracellular environment, as well as the interaction of regulatory factors, DNMT3B can play both a promoting and inhibitory role in the osteogenic differentiation of MSCs. DNMT3B mediated hypermethylation of the Galactin-1 gene (Gal-1) promoter plays an important role in the downregulation of Gal-1 in elderly mouse BMSCs. This inhibits β-catenin binding to the Gal-1 promoter gene, reduces Gal-1 expression, impairs the function of mouse BMSCs, and inhibits their osteogenic differentiation (Tang et al., 2024). During mechanical unloading in mice, the protein level of DNMT3B increases. This leads to the methylation of the Sonic hedgehog (Shh) gene promoter, which causes the downregulation of Shh. This process inhibits the Hedgehog (Hh) signaling pathway, impairs the osteogenic differentiation ability of mouse BMSCs (Wang et al., 2017). Fork shaped box C1 (FOXC1) can target DNMT3B and promote its expression. DNMT3B methylates the promoter of C-X-C chemokine ligand 12 (CXCL12), which inhibits the expression of CXCL12. As a result, this process promotes osteogenic differentiation of hBMSCs (Zhang et al., 2024). hBMSCs exhibit lower phosphatase and tensin homolog (PTEN) expression and lower osteogenic potential compared to human dental pulp mesenchymal stem cells (hDPMSCs) due to the high methylation of DNMT3B-mediated PTEN gene promoters (Shen et al., 2019).

DNMT3L is a protein that does not have enzymatic activity but plays a crucial role in promoting *de novo* DNA methylation. It interacts with DNMT3A and DNMT3B to affect gene expression. However, its role in regulating MSCs osteogenic differentiation is not well studied. In a study by Chih Yi Yang et al., it was found that DNMT3L protein is not expressed in cultured BMSCs. They also discovered that knockout mice derived BMSCs (DNMT3L-KO-MSCs) have impaired *in vitro* osteogenesis and reduced colony forming ability. It was observed that the osteogenic differentiation ability is linked to the pre-deposition epigenetic characteristics of DNMT3L expressing progenitor cells (Yang et al., 2021).

## 4 The role of TETs in osteogenic differentiation of MSCs

Mammalian TETs such as TET1, TET2, and TET3, are responsible for DNA demethylation, which can regulate gene expression by opening up chromatin for transcription regulators (Chakraborty et al., 2022). TETs are expressed in various cell lineages and play a crucial role in maintaining MSCs homeostasis and regulating their lineage differentiation (Yu et al., 2019; Alicka et al., 2020) (Table 3). TETs mediated DNA demethylation is essential for maintaining embryonic bone formation. Knocking out TET1/2/3 in mesenchymal stem cells under Prx1 Cre conditions can lead to severe defects in bone development, including delayed closure of the fontanelle, clavicular hypoplasia, and limb shortening. However, any allele that retains the TETs gene can maintain bone formation, indicating the functional redundancy of TETs in bone development (Wang L. et al., 2022).

**TABLE 3:** The role of TETs in osteogenic differentiation of MSCs.

TETs type	Cell type	Brief outcomes	The effect of TETs on osteogenic differentiation potential	References
TET1	mouse BMSCs	TETs↓→5 hmC↓→osteogenic differentiation↓	↑	Glemžaitė and Navakauskienė (2016)
	human DPSCs	three-dimensional spheres →TET1↑→osteogenic differentiation↑	↑	Hyslop et al. (2005)
	mouse BMSCs	TET1, TET2↓→P2rX7↓→osteogenic differentiation↓	↑	Okano et al. (1998)
	human BMSCs	TET1 recruits SIN3, EZH2→ osteogenic genes↓→osteogenic differentiation↓	↓	Ornitz and Marie (2002)
	human BMSCs	TET2, TET1→ osteogenic genes↑→osteogenic differentiation↑	↑	Ornitz and Marie (2002)
TET2	mouse BMSCs	TETs↓→5 hmC↓→osteogenic differentiation↓	↑	Glemžaitė and Navakauskienė (2016)
	human ADSCs	TET2, TET3↑→5 hmC↑→osteogenic differentiation↑	↑	Chakraborty et al. (2022)
	human ADSCs	TET2↓→5 hmC↓→osteogenic genes↓→osteogenic differentiation↓	↑	O'Hagan et al. (2011)
	human BMSCs	miR-144-3p→TET2↓→5 hmC↓→osteogenic gene↓→osteogenic differentiation↓	↑	Okano et al. (1999)
	mouse BMSCs	TET1, TET2↓→P2rX7↓→osteogenic differentiation↓	↑	Okano et al. (1998)
	PDLSCs	TET2 combines with HDAC1→E-cadherin↓→osteogenic differentiation↑	↑	Oliveira et al. (2012)
	mouse BMSCs	TET2↓→dysregulation of 5 hmC→ altered expression of osteogenic genes →osteogenic differentiation↑	↓	Ooi and Bestor (2008)
	human BMSCs	TET2, TET1→osteogenic genes↑→osteogenic differentiation↑	↑	Ornitz and Marie (2002)
TET3	mouse BMSCs	TETs↓→5 hmC↓→osteogenic differentiation↓	↑	Glemžaitė and Navakauskienė (2016)
	human ADSCs	TET2, TET3↑→5 hmC↑→osteogenic differentiation↑	↑	Chakraborty et al. (2022)

Note: ↑: Promoting; ↓: Inhibiting; →: Regulating.

Abbreviation: TETs, Ten Eleven Translation Family Proteins; MSCs, mesenchymal stem cells; TET1, Tet methylcytosine Dioxygenase 1; TET2, Tet methylcytosine Dioxygenase 2; TET3, Tet methylcytosine Dioxygenase 3; BMSCs, bone marrow mesenchymal stem cells; 5 hmC, 5-hydroxymethylcytosine; ADSCs, Adipose Derived Stem Cells; DPSCs, Dental pulp stem cells; P2rX7, Purinergic Receptor P2X 7; PDLSCs, Periodontal Ligament Stem Cells; HDAC1, Histone Deacetylase 1; EZH2, Enhancer Of Zeste 2 Polycomb Repressive Complex 2 Subunit.

TETs are molecules that play a crucial role in promoting the process of osteogenic differentiation of MSCs. They are especially important in promoting the differentiation of MSCs into bone cells. The osteogenic potential of hADSCs from elderly donors is improved by an increase in 5hmC levels and increased expression of TET2 and TET3 genes (Yan et al., 2014). During the process of osteogenic differentiation of hADSCs, TET2 is upregulated. Inhibition of TET2 may decrease the expression of osteogenic-related genes and downregulate 5hmC, which can ultimately have a negative effect on osteoporosis (Feng et al., 2020). In patients with aplastic anemia, low expression of TET2 in hBMSCs leads to a decrease in overall 5hmC levels, inhibiting osteogenic differentiation (Li N. et al., 2020). However, hDPSCs derived from three-dimensional sphere culture (3D) are more inclined to differentiate into osteogenic lineage than two-dimensional monolayer culture (2D) hDPSCs. TET1 mRNA transcripts are significantly upregulated in hDPSCs cultured in three-dimensional spheres, and TET1 plays a crucial role in the osteogenic differentiation of hDPSCs cultured in three-dimensional spheres (Raik et al., 2022). The demethylation of the P2rX7 promoter in BMSCs of TET1 and TET2 knockout mice is inhibited, resulting in impaired osteogenic differentiation of mouse BMSCs (Yang et al., 2018). Finally, the hydrogel system developed by Tingting Yu et al. showed an upregulation of TET2 in PDLSCs. TET2 combined with histone deacetylase 1(HDAC1) to inhibit the transcriptional expression of E-cadherin and promote the osteogenic differentiation of PDLSCs (Yu T. et al., 2022).

Some studies have suggested that TETs may impair the osteogenesis process of MSCs. For instance, TET2 knockout mouse BMSCs have shown dysregulation of 5mC hydroxymethylation and altered expression of key genes related to osteogenic differentiation, thereby exhibiting enhanced osteogenic differentiation potential, cell proliferation, and self-renewal (Li et al., 2018). However, other research has suggested that TETs can both inhibit and promote osteogenic differentiation of MSCs. Research results have shown that TET1 in hBMSCs inhibits osteogenic gene transcription by recruiting SIN3A and EZH2, thereby inhibiting osteogenic differentiation, while TET2 leads to low methylation of osteogenic genes and upregulation, promoting osteogenic differentiation. TET1 also plays a role in this process (Cakouros et al., 2019). Although TETs have some inhibitory effect on the osteogenic differentiation of MSCs, they mainly exhibit a promoting effect. However, this situation may be influenced by factors such as the gene regulatory network it participates in, epigenetic modifications, and intracellular environment.

## 5 The role of DNA methylation in the osteogenic differentiation signaling pathway of MSCs

The process of MSCs differentiation involves two steps: lineage shaping (from MSCs to lineage-specific progenitor cells) and maturation (from progenitor cells to specific cell types) (Chen Y. S. et al., 2016). Before directly differentiating into bone cells, MSCs tend to differentiate into osteoblasts. Pre-osteoblasts then develop into mature osteoblasts, which synthesize bone matrix and embed bone cells into it (James, 2013). Multiple signaling pathways play a role in regulating the osteogenic differentiation of MSCs, such as Notch (Fiddes et al., 2018), Hedgehog signaling (Carballo et al., 2018), Bone Morphogenetic Protein (BMP)—Smad (Wang et al., 2014), Wnt signaling (Monroe et al., 2012), Mitogen-activated protein kinase (MAPK) (Shukla et al., 2007), Fibroblast growth factor (FGF) (Ornitz and Marie, 2002), Ras homologous gene family member A/Rho kinase (RhoA/ROCK) (McBeath et al., 2004), and Neuroepidermal growth factor like protein 1 (NELL-1) (Li C. et al., 2019). These signaling pathways interact to form a complex regulatory network that regulates the osteogenic differentiation of MSCs. As targets of these signaling pathways, Runx2 and Osterix are important transcription factors that play a crucial role in the process of osteogenic differentiation of MSCs. In addition to Runx2 and Osterix, there are other transcription factors such as Dlx5, Dlx3, FRA1, Twist1, ZBTB16, and ATF4 that also participate in osteoblast differentiation (Harada and Rodan, 2003; Yang et al., 2004). Once osteogenic signals are activated in pre-osteoblasts, these signaling pathways regulate the expression and function of major transcription factors in osteoblasts. This helps control the expression of downstream bone phenotype genes, ultimately establishing the osteoblast components of mammalian bones (Maroni et al., 2012).

**FIGURE 4:**
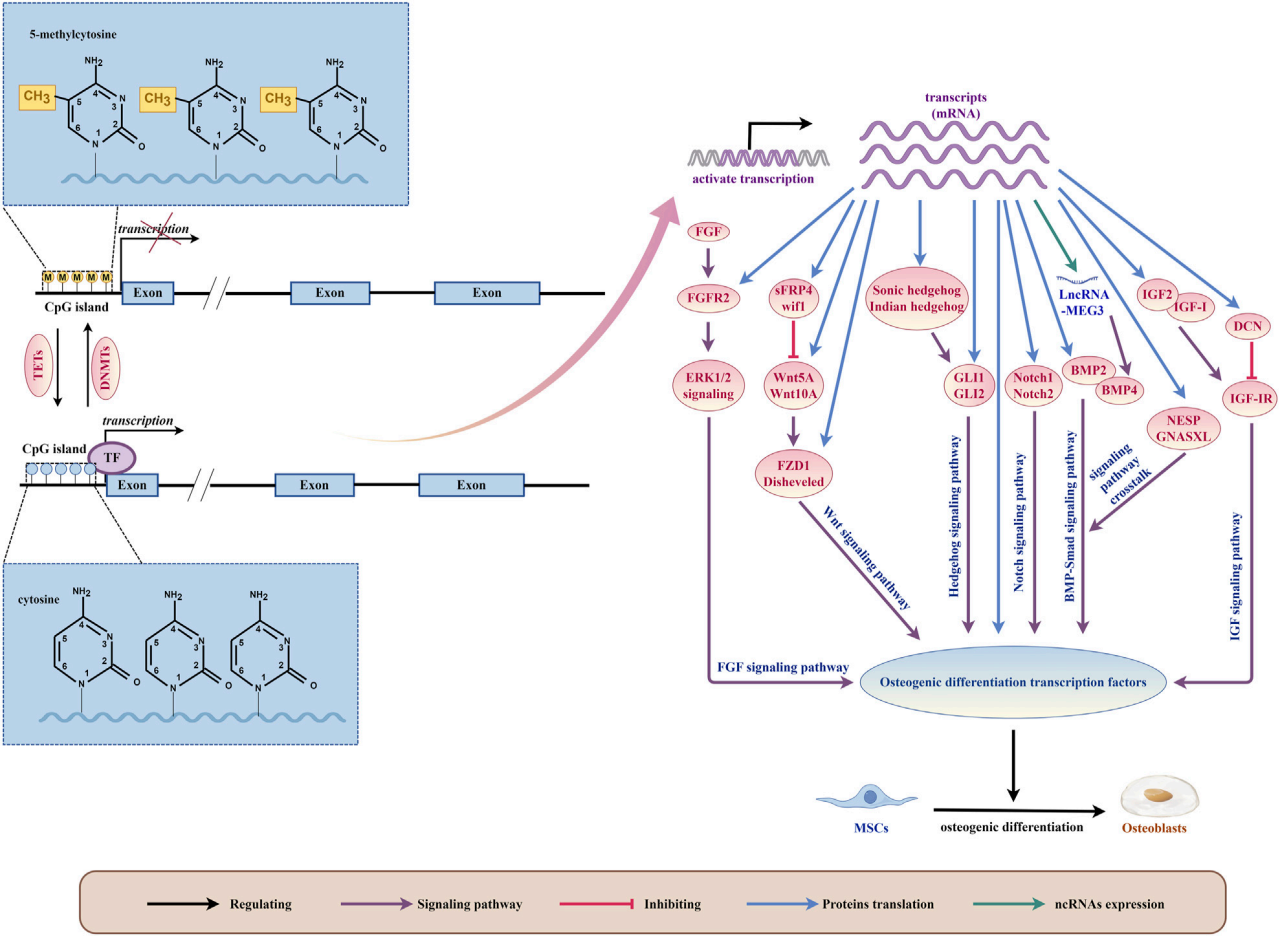
DNA methylation plays a critical role as a regulatory mechanism in orchestrating the osteogenic differentiation of bone marrow mesenchymal stem cells (MSCs) by modulating intricate signaling pathways and osteogenic differentiation transcription factors. (By Figdraw.). This biochemical phenomenon occurs specifically at the 5mC site on the DNA strand, catalyzed by DNA methyltransferases (DNMTs). Conversely, the action of Ten-eleven translocation family proteins (TETs) leads to the oxidation of 5mC to 5hmC, thereby facilitating active demethylation of DNA. This demethylation process dynamically influences gene transcription, thereby enabling the translation of diverse signaling pathway components pivotal for regulating osteogenic transcription factors essential for MSCs’ osteogenic differentiation. Furthermore, transcripts themselves can directly translate osteogenic differentiation transcription factors, further intricately fine-tuning the regulatory machinery governing MSCs’ osteogenic fate determination.

The impact of DNA methylation on the differentiation of MSCs towards bone-forming cells is complex and multi-faceted (Figure 4). Studies have shown that DNA methylation can regulate the expression, activity, and interaction of certain transcription factors, which in turn affect MSCs differentiation (Farshdousti Hagh et al., 2015; Marofi et al., 2019a; Marofi et al., 2019b; Fang et al., 2019; Marofi et al., 2020). Additionally, DNA methylation can also influence the activity of signaling pathways by regulating the expression or methylation status of genes involved in these pathways, ultimately affecting the osteogenic differentiation of MSCs (Table 4).

**TABLE 4:** The role of DNA methylation in the osteogenic differentiation signaling pathway of MSCs.

Gene loci of DNA methylation	Cell type	Brief outcomes	References
Wnt5A	human MSCs	hMSCs separated from spinal ligaments with ectopic ossification tend to have an osteogenic lineage, which is related to low methylation of the Wnt5A gene	Tang et al. (2024)
Wnt10A	ST2 cells	The expression of Wnt10A is influenced by its CpG site methylation regulation, thereby regulating the lineage of adipogenesis and osteoblast generation	Tao et al. (2022)
Disheveled	BMSCs	The degree of methylation in the promoter region of the Disheveld gene can affect the expression of Disheveld and the expression of Wnt signaling molecules, thereby affecting osteogenic differentiation	Thomas and Jaganathan (2022)
FZD1	human BMSCs	hBMSCs in patients with femoral head necrosis exhibit abnormal CpG island hypermethylation in FZD1, with lower transcription and translation levels, leading to reduced osteogenic ability	Tsai et al. (2012)
Wif1	human BMSCs	Dynamic downregulation of Wif1 gene is associated with maintaining methylation of gene promoter during different stages of osteogenic differentiation in hBMSCs	Urakami et al. (2006)
sFRP4	mouse BMSCs	KDM4A directly combines sFRP4 and C/EBP α Reduced methylation of their promoter regions leads to sFRP4 and C/EBP α Increased expression, thereby blocking osteogenic differentiation	Veland et al. (2019)
sFRP4	ST2 cells	Oxidative stress-induced guanine modification relieves the inhibition of sFRP4 gene by isolating MeCP2 and recruiting TBP at the TATA box in reverse, leading to impaired osteogenic differentiation of mouse bone marrow-derived ST2 cells	Vlaikou et al. (2017)
Notch1, Notch2	human ADSCs	Abnormal DNA methylation of Notch1 and Notch2 leads to transcriptional upregulation and osteogenic differentiation of hADSCs	Li et al. (2021a)
Notch1	human BMSCs	The methylation level of Notch1 promoter increases, leading to a decrease in Notch1 expression and impaired osteogenic differentiation of BMSCs in patients with cyanotic congenital heart disease	Wang et al. (2022b)
Notch1	mouse BMSCs	Low methylation of Notch1 promoter and activation of Notch signaling in lupus disease mice lead to impaired osteogenic differentiation	Kato et al. (2009)
Sonic hedgehog	mouse BMSCs	Mechanical unloading in mice leads to methylation of the Sonic hedgehog gene promoter and downregulation of Sonic hedgehog, impairing osteogenic differentiation ability	Montecino et al. (2020)
Indian hedgehog	mouse C3H10T1/2 cells	Demethylation of Indian Hedgehog promoter in mouse C3H10T1/2 mesenchymal stem cells improves osteogenic differentiation ability	Jiang et al. (2019)
Sonic hedgehog, GLI1, GLI2	human BMSCs	The carcinogenic metabolite R-2HG induces significant DNA hypermethylation in hBMSCs, reducing the mRNA levels of Sonic hedgehog, GLI1, and GLI2, and blocking osteogenic differentiation	Wu et al. (2019)
BMP2	human eMSCs	Lovastatin treatment induces demethylation of the BMP2 promoter, increases the expression of BMP2 and RUNX2, and promotes osteogenic lineage differentiation of human eMSCs	Yan et al. (2014)
BMP2	human PDLSCs	5-aza-Dc increases BMP-2 hydroxymethylation levels, increases BMP-2 transcriptional expression, and leads to osteogenic differentiation	Yang et al. (2021)
BMP4	human BMSCs	The high methylation of the LncRNA-MEG3 gene promoter inhibits the expression of LncRNA-MEG3, leading to downregulation of BMP4 transcriptional activity and impairment of osteogenic differentiation	Kim et al. (2013b)
GNAS	human ADSCs	Mechanical unloading leads to demethylation and upregulation of key CpG sites in the GNAS subtype NESP and GNASXL of hADSCs, promoting early osteogenic differentiation, which is related to crosstalk in the BMP signaling pathway	Youssef and Han (2017)
DCN	human BMSCs	The expression level of DCN gene is positively correlated with CpG methylation of this gene, thereby inhibiting IGF-IR to impair IGF-I signaling and osteogenic differentiation	Yu et al. (2022b)
IGF2	human SHEDs	The methylation of the imprint control region within the IGF2-H19 locus in human SHED increased by 4%. The upregulation of IGF2 in SHED is associated with the loss of imprints, which gives it higher osteogenic potential compared to human adipose derived mesenchymal stem cells	Zhang et al. (2016)
FGFR2	human MSCs	Early nickel exposure induces hypomethylation of the FGFR2 promoter, increasing its binding affinity with transcription factor Sp1, leading to FGFR2 activation. FGFR2 is a key regulatory factor in ERK1/2 signaling, thereby promoting osteogenic differentiation of hMSCs through ERK1/2 signaling	Zhu et al. (2014)

Abbreviation: MSCs, mesenchymal stem cells; Wnt5A, Wnt family member 5A; hMSCs, human mesenchymal stem cells; Wnt10A, Wnt family member 10A; BMSCs, bone marrow mesenchymal stem cells; FZD1, Frizzled Class Receptor 1; hBMSCs, human bone marrow mesenchymal stem cells; Wif1, Wnt Inhibitory Factor 1; sFRP4, Secreted Frizzled Related Protein 4; KDM4A, Lysine Demethylase 4A; C/EBP α, CCAAT, enhancer binding protein alpha; MeCP2, Methyl-CpG, Binding Protein 2; TBP, TATA-Box Binding Protein; ADSCs, Adipose Derived Stem Cells; hADSCs, human Adipose Derived Stem Cells; GLI1, GLI, Family Zinc Finger 1; GLI1, GLI, Family Zinc Finger 2; R-2HG, R-2-Hydroxyglutarate; BMP2, Bone Morphogenetic Protein 2; eMSCs, endometrium-derived mesenchymal stem cells; RUNX2, RUNX, Family Transcription Factor 2; PDLSCs, Periodontal Ligament Stem Cells; DCN, decorin; IGF-IR, Insulin Like Growth Factor 1 Receptor; IGF-I, Insulin Like Growth Factor 1; IGF2, Insulin Like Growth Factor 2; SHEDs, human deciduous teeth mesenchymal stem cells; FGFR2, Fibroblast Growth Factor Receptor 2.

### 5.1 DNA methylation regulates osteogenic differentiation of MSCs through the Wnt signaling pathway

The Wnt signaling pathway is a crucial factor in promoting the osteogenic differentiation of MSCs. The classical Wnt pathway inhibits the primary lipid inducer, PPAR γ Binding protein, with C/EBP alpha. This inhibition prevents adipogenic differentiation and upregulates osteogenic regulatory factors such as Runx2, Dlx5, and Osterix, which promotes osteogenic differentiation. Furthermore, non-classical Wnt pathways induce osteogenic differentiation through different mechanisms (Kim J. H. et al., 2013). During the osteogenic differentiation process of MSCs, a whole-genome DNA methylation analysis revealed the enrichment of the Wnt signaling pathway, demonstrating its critical role in the osteogenic differentiation process of MSCs (Del Real et al., 2017; Cao et al., 2018; Lyu et al., 2019).

As discussed in the previous section of this review, AGEs can negatively impact the ability of MSCs to differentiate into bone cells and promote the formation of bone-dissolving cells. When rat and mouse ADSCs were grown in a medium containing AGEs, it caused an increase in global 5-mC, inhibited the classic Wnt signaling pathway, and hindered osteogenic differentiation (Zhang et al., 2018; Li Y. et al., 2020). In mice with diabetes-induced osteoporosis, the mesenchymal stem cells derived from adipose tissue (DOP ADSCs) had hypermethylation of genes related to osteogenesis and the Wnt/β-catenin signaling pathway, which reduced the activity of the pathway and impaired the osteogenic differentiation of DOP ADSCs (Zhang M. et al., 2022).

DNA methylation plays a significant role in regulating the Wnt signaling pathway during the osteogenic differentiation process of MSCs. This process affects the expression and activity of the components of the Wnt signaling pathway. When isolated from spinal ligaments with ectopic ossification, human mesenchymal stem cells (hMSCs) tend to have an osteogenic lineage, which is associated with low methylation of the Wnt5A gene (Chiba et al., 2015). The Wnt10A gene is a crucial factor that determines the fate of mesenchymal stem cells towards osteoblasts. Its 5′-region is rich in CpG sites, and its expression can be influenced by methylation regulation of these sites, which in turn regulates the lineage determination of MSCs, such as adipogenesis and osteoblast generation (Chen Q. et al., 2016). As the differentiation of osteoblasts increases, the expression of Disheveled also increases. The regulation of the degree of methylation in the Disheveled gene promoter region can affect the expression of Disheveled, which can then affect the expression of Wnt signaling molecules and, thus, affect the osteogenic differentiation of BMSCs (Han et al., 2017). Patients with femoral head necrosis have hBMSCs and Wnt signaling pathway receptor FZD1 with abnormal CpG island hypermethylation, leading to low transcription and translation levels. As a result, the Wnt/β-catenin signaling pathway is inhibited, and the osteogenic ability of hBMSCs is weakened, while their ability to generate fat is enhanced (Wu et al., 2019).

The Wnt antagonist family, which includes sFRP (secreted curl related protein), WIF-1 (Wnt inhibitor 1), Cerberus, and Dickkopf (Dkk), affect the transmission of the Wnt signaling pathway in various ways (Urakami et al., 2006). DNA methylation indirectly influences the Wnt signaling pathway by regulating the expression, activity, and interaction of regulatory factors with Wnt signaling pathway antagonists, thereby affecting the osteogenic differentiation of MSCs. During different stages of osteogenic differentiation in hBMSCs, dynamic downregulation of the Wif1 gene is related to the maintenance of methylation of the gene promoter (Mashhadikhan et al., 2020). Histone lysine demethylase 4A (KDM4A) directly binds to sFRP4 and C/EBP alpha, reducing the DNA methylation level of CpG in the promoter region of the C/EBP alpha and sFRP4 promoters, increasing the expression of C/EBP alpha and sFRP4, inactivating classical Wnt signaling, promoting adipogenic differentiation of mouse BMSCs, and blocking osteogenic differentiation (Qi et al., 2020). Oxidative stress-induced guanine modification relieves the inhibition of the sFRP4 gene by isolating MeCP2 and instead recruiting TBP at the TATA box. This inhibits Wnt signaling and impairs osteogenic differentiation of mouse bone marrow stromal derived ST2 cells (Mori et al., 2014).

### 5.2 DNA methylation regulates osteogenic differentiation of MSCs through the Notch signaling pathway

The Notch signaling pathway is a pathway that has been conserved throughout evolution and is responsible for regulating cell proliferation and fate determination in both embryonic and adult organs. This pathway plays an important role in maintaining the stemness of multiple stem cells and guiding differentiation (Boni et al., 2008). The Notch signaling pathway has been extensively studied in bone formation and remodeling. It helps to maintain the bone marrow mesenchymal progenitor cell bank and regulates osteoclastogenesis by directly or indirectly regulating osteoblasts (Engin et al., 2008).

*In vitro* cell culture experiments have shown that activating Notch signaling can have both promoting and inhibiting effects on osteoblast differentiation and mineralization, while inhibiting Notch signaling can also have promoting or inhibiting effects (Ji et al., 2017). However, the contradictory role of the Notch signaling pathway in bone formation appears to be influenced by the DNA methylation process that regulates the osteogenic differentiation of MSCs. Abnormal DNA methylation of Notch1 and Notch2 can lead to transcriptional upregulation, promoting osteogenic differentiation of hADSCs (Jia et al., 2021). On the other hand, increased methylation levels of the Notch1 promoter result in a decrease in Notch1 expression, which can lead to poor multi-lineage differentiation potential of hBMSCs in patients with cyanotic congenital heart disease (CCHD) and impaired osteogenic differentiation of hBMSCs (Wang et al., 2020). In lupus disease mice, low methylation levels of the Notch1 promoter and activation of Notch signaling have been found to lead to impaired osteogenic differentiation of BMSCs (Liu et al., 2015). The contradictory role of Notch in osteogenesis can be explained by considering the relationship between the Notch signaling pathway effect in bones and the cellular environment. Specifically, when Notch is expressed in immature osteoblasts, it can prevent their differentiation, leading to bone loss. Conversely, when Notch is expressed in bone cells, it can initially inhibit bone resorption and increase bone mass (Canalis et al., 2013; Shao et al., 2018).

### 5.3 DNA methylation regulates osteogenic differentiation of MSCs through the hedgehog signaling pathway

The Hedgehog signaling (Hh) pathway is highly evolved and plays important roles in embryonic and bone development. Research has shown that Hh signaling positively regulates the differentiation of MSCs into osteoblasts (James et al., 2010; Kim et al., 2010; Jiang et al., 2019). This is achieved by affecting the expression of two important transcription factors, RUNX2 and Osterix (Oliveira et al., 2012).

Hedgehog proteins, which include Sonic Hedgehog (SHH), Indian Hedgehog (IHH), and Desert Hedgehog (DHH), exist in mammals. When the extracellular Hedgehog protein binds to the transmembrane receptor (Ptch), it releases the inhibition of a specific labeled smooth receptor (Smo) and further phosphorylates it. This activation of Smo releases the Hedgehog pathway transcriptional effector Ci/Gli from Cos2 and transfers it to the nucleus, activating the expression of the corresponding downstream target genes (Petrova and Joyner, 2014). Overexpression of SHH significantly increases the osteogenic ability of rat BMSCs, both *in vitro* and *in vivo* (Jiang et al., 2019). However, mechanical unloading in mice leads to methylation of the SHH gene promoter and downregulation of SHH, inhibiting the Hh pathway and impairing the osteogenic differentiation ability of mouse BMSCs (Wang et al., 2017). IHH upregulates the expression of RUNX2 and osteogenesis through GLI2, thereby stimulating the differentiation of BMSCs into osteoblasts (Shimoyama et al., 2007). Overexpression of GLI1 can reverse oxidative stress-mediated reduction in the osteogenic differentiation of mouse MSCs (Kim et al., 2010). Studies have shown that demethylation of the IHH promoter in mouse C3H10T1/2 mesenchymal stem cells improves their osteogenic differentiation ability (Tao et al., 2022). The carcinogenic metabolite R-2HG induces a significant DNA hypermethylation state in hBMSCs, inducing high methylation and reducing mRNA levels of SHH, GLI1, and GLI2. Consequently, SHH signaling is inhibited, blocking the osteogenic differentiation of hBMSCs while promoting adipose differentiation of hBMSCs (Liu et al., 2021).

### 5.4 DNA methylation regulates osteogenic differentiation of MSCs through the BMP-Smad signaling pathway

BMP is a type of pleiotropic cytokine that belongs to the transforming growth factor-β (TGF-β) superfamily. BMP signal transduction occurs through type I and type II BMP receptors. When BMP ligands bind to these receptors, homodimers of type II receptors form a tetrameric complex with homodimers of type I receptors. This leads to the phosphorylation of type I receptors, resulting in signal transduction through Smads. This dynamic interaction activates the transcription of specific target genes involved in osteoblast differentiation and bone formation (Wu et al., 2016).

Overexpression of bone morphogenetic protein 2 (BMP2) promotes the differentiation of BMSCs into osteogenic cells. The osteogenic cell line differentiation is believed to be stimulated by the BMP-2 receptor IB, which subsequently activates various transcription factors such as Runx2 and Osterix (Kato et al., 2009; Davis et al., 2011). Lovastatin treatment induces demethylation of the BMP2 promoter, which increases the expression of BMP2 and RUNX2, and promotes the osteogenic lineage differentiation of human endometrial mesenchymal stem cells (eMSCs) (Taghizadeh and Noruzinia, 2017). In human periodontal ligament CD105^+^ MSCs, 5-aza-dC demethylates BMP2 by increasing the level of hydroxymethylation, which in turn increases BMP2 transcriptional expression. BMP2 signaling molecules activate the phosphorylation and nuclear translocation of Smad protein, which interacts with RUNX2, leading to upregulation of ALP and OCN transcripts. This results in osteoblast differentiation (Sacramento et al., 2022). BMP4 has the potential to regulate the osteogenic differentiation of MSCs. After transfection of BMP4 gene into MSCs of patients with aplastic anemia (AA), the adipogenic differentiation ability is reduced, while the osteogenic differentiation is enhanced (Cheng et al., 2015). High methylation of the promoter of the LncRNA MEG3 gene in children with aplastic anemia (AA) inhibits the expression of LncRNA MEG3. Subsequently, low expression of LncRNA MEG3 leads to downregulation of BMP4 transcriptional activity, impairing the osteogenic differentiation of BMSCs in AA patients (Li H. et al., 2021).

Studies have demonstrated that the dynamic regulation of GNAS and cAMP levels, along with the crosstalk with BMP signaling pathways, are also associated with the initiation and progression of osteoblast differentiation (Zhang et al., 2012). The application of mechanical stress leads to the demethylation and upregulation of critical CpG sites in the GNAS subtype NESP and GNASXL of hADSCs, which promotes early osteogenic differentiation (Vlaikou et al., 2017). This demethylation and upregulation of key CpG sites may be linked to crosstalk in the BMP signaling pathway.

### 5.5 DNA methylation regulates osteogenic differentiation of MSCs through the IGF signaling pathway

Insulin-like growth factors (IGFs), including insulin-like growth factor 1 (IGF-1) and insulin-like growth factor 2 (IGF-2), have the ability to stimulate and promote the differentiation of stem cells into various lineages in all three embryonic layers. This includes the differentiation of osteoblasts and bone development *in vivo*. IGF-1, IGF-2, and their receptor type 1 insulin-like growth factor receptor (IGF-1R) are closely associated with osteogenesis (Youssef and Han, 2017).

The ability of BMSCs to stimulate cell growth (mitogenic activity) through IGF-I is reduced in elderly donors due to aging. However, the overexpression of IGF-I can boost the proliferation and bone formation ability of BMSCs from elderly donors (Chen et al., 2017). During the aging process of hBMSCs, the expression level of the decorin (DCN) gene is positively related to the CpG methylation in the second intron of the gene. This methylation inhibits the IGF-I signaling through IGF-IR, which impairs the osteogenic differentiation potential of hBMSCs (Wong et al., 2020).

The imprinting region of the IGF2 gene is controlled by the H19DMR (differentially methylated region) gene, which plays a crucial role in parental-specific silencing of the adjacent H19 and IGF2 genes (Reik et al., 2001). IGF2 is crucial for the differentiation of MSCs into osteoblasts. During the process of osteogenic differentiation, IGF2 and IGF binding protein 2 (IGFBP2) are upregulated (Karasik et al., 2002), which also occurs in dental mesenchymal stem cells (Morsczeck et al., 2010). The methylation of the imprint control region within the IGF2-H19 locus in human deciduous teeth mesenchymal stem cells (SHED) increases by 4%. The upregulation of IGF2 in SHED is associated with the loss of imprints, which gives it higher osteogenic potential compared to hADSCs (Fanganiello et al., 2015).

### 5.6 DNA methylation regulates osteogenic differentiation of MSCs through the FGF signaling pathway

Fibroblast Growth Factor (FGF) works by binding to fibroblast growth factor receptors (FGFRs) present on cell surfaces. This binding triggers a series of complex downstream signaling pathways, including the PLCγ pathway, the RAS-MAP kinase pathway involving ERK1/2, p38, and JNK kinases, and the PI3K/AKT pathway (Moosa and Wollnik, 2016). FGF signaling plays a crucial role in maintaining bone homeostasis, and the level of FGF receptor 2 (FGFR2) is linked to the osteogenic potential of hMSCs *in vitro* (Zhang Y. et al., 2022).

Mutations in any of the three members of the FGFRs family (FGFR1, FGFR2, and FGFR3) can lead to an imbalance in FGF signaling and cause premature closure of cranial sutures (Azoury et al., 2017). Studies suggest that nickel exposure at an early age can reduce the methylation of the FGFR2 promoter, which enhances its affinity for the transcription factor Sp1. This leads to the activation of FGFR2, which plays a vital role in regulating ERK1/2 signaling. Consequently, osteogenic differentiation of hMSCs is promoted through ERK1/2 signaling, which results in cranial suture fusion (Weng et al., 2024).

## 6 Conclusion and perspective

DNA methylation is a crucial biological process that regulates gene transcription and plays a vital role in various cellular activities, including stem cell fate determination, and cell differentiation. MSCs are self-renewing cells with the potential for multiple types of cell differentiation, making them a highly promising candidate for tissue engineering and regenerative medicine applications.

Current research suggests that DNA methylation plays a critical role in the osteogenic differentiation of MSCs. However, directing the osteogenic lineage differentiation of MSCs and utilizing their unique properties for bone tissue engineering represents a significant challenge. Due to variations in their osteogenic differentiation potential among different tissue sources, MSCs derived from diverse sources cannot be used for therapeutic purposes. Although some MSCs, such as BMSCs, have higher osteogenic efficiency, their limited availability and discomfort associated with obtaining them limit their widespread use. Future research should focus on enhancing the osteogenic differentiation potential of MSCs through DNA methylation mechanisms or developing novel strategies for osteogenic differentiation therapy of bone defect-related MSCs based on DNA methylation.

MSCs have been studied extensively and used successfully in bone tissue engineering and clinical therapy under *in vitro* culture conditions. However, various factors *in vivo* could impair their osteogenic differentiation, and MSCs need to be guided to the corresponding sites *in vivo* to exert their effects. Therefore, the study of DNA methylation in the *in vivo* osteogenic differentiation of MSCs and their directional migration *in vivo* is particularly crucial.

By identifying the target genes regulated by DNA methylation during MSCs osteogenic differentiation, we can develop novel epigenetic drugs using new drug screening techniques and drug delivery methods. The development of such epigenetic drugs is expected to bring new therapeutic strategies and drug choices to the field of orthopedics, potentially improving bone tissue regeneration and treatment outcomes for patients.
